# Osteomyelitis in Late-Stage Pressure Sore Patients: A Retrospective Analysis

**DOI:** 10.3390/life14080973

**Published:** 2024-08-02

**Authors:** Marc Ruewe, Andreas Siegmund, Markus Rupp, Lukas Prantl, Alexandra M. Anker, Silvan M. Klein

**Affiliations:** 1 Department of Plastic, Hand and Reconstructive Surgery, University Hospital Regensburg, 93053 Regensburg, Germany; 2 Department of Trauma Surgery, University Hospital Regensburg, 93053 Regensburg, Germany

**Keywords:** pressure sore, pressure ulcer, reconstructive surgery, tissue repair, patient outcomes, humans, aged, retrospective analysis, osteomyelitis, c-reactive protein, blood count

## Abstract

Background: Late-stage pressure sore (PS) patients are particularly susceptible to osteomyelitis (OM), as bony prominences commonly constitute the focal point of the ulcer. There are lack of data regarding the associated factors and the clinical relevance of this diagnosis in the context of PS treatment. Methods: This retrospective analysis investigated the clinical characteristics, blood markers indicative of infection in PS patients, and development of histologically evident OM. A total of 125 patient were included from 2014 to 2019. The patient records were especially scanned for histological diagnosis of OM. Results: OM was detected in 39% (37/96) of the samples taken during the index procedure. OM prevalence increased to 56% (43/77) at the second and 70% (41/59) at the third debridement. Therefore, the diagnosis of OM was acquired during treatment in 35 cases. Patients diagnosed with initial OM presented significantly higher blood markers, indicative of infection upon admission. Only patients with consistent OM (three positive biopsies) showed higher flap revision rates. Conclusion: This study found no compelling evidence linking OM to worse clinical outcomes in PS patients. In the absence of elevated inflammatory markers, reducing bone biopsy frequency and adopting a less aggressive bone debridement approach may help prevent OM in PS patients.

## 1. Introduction

Pressure sores (PS), also known as pressure ulcers, are localized ischemic injuries to soft tissue caused by obstructed blood flow resulting from constant mechanical force typically on bony prominences. This condition is a common and severe complication in immobile patients, with estimated total costs of USD 11 billion per year in the US [1]. Current guidelines suggest the classification of PS according to the level of tissue injury into four stages [2,3]. While the first three stages involve no exposure of deeper anatomical structures, stage four PS consist of wounds with extensive tissue destruction and frequently exposed bone. Stages one and two exhibit sufficient healing potential if prompt pressure relief and proper skin care is provided. Stages three and four typically necessitate surgical removal of necrotic tissue through debridement, followed by subsequent soft tissue coverage. 

Based on the specific route of bacterial invasion, two types of bone infections can be distinguished, each requiring tailored therapeutic strategies. Hematogenous osteomyelitis (OM) may originate from bacterial infection associated with the bloodstream, whereas exogenous OM typically arises from the contamination of injured exposed bone structures [4]. Thus, late-stage PS patients are particularly susceptible to exogenous OM, as bony prominences commonly constitute the focal point of the ulcer. 

Large wound areas, prolonged wound healing phases, and the proximity of the lesion to the anus, combined with a generally compromised immune defense in these patients, exacerbate bacterial load on the wound surface and contributes to the deep infection of the affected tissue [5]. Therefore, serial debridement, routinely in conjunction with temporary negative pressure wound therapy, precede reconstructive efforts in surgery to reduce the bioburden prior to the final closure of chronic wounds [6,7,8]. Surgical curing of OM is generally complicated, as formation of bony sequestra may necessitate extended debridement, which include additional surgical challenges such as a tendency for uncontrolled bleeding, poor accessibility, and high morbidity associated with extensive bone loss [4,9,10]. Moreover, the limited capacity of numerous antibiotics to penetrate bone results in diminished tissue concentrations, thereby fostering the potential for bacterial resistance and ultimately culminating in the failure of bacterial eradication [11,12]. Existing evidence on the management of PS patients is notably controversial. Some studies advocate for a multiple debridement strategy to prevent treatment failure, while others discourage multistep debridement protocols before reconstruction, seemingly without adverse effects on clinical outcomes [13,14,15,16,17]. Additionally, several studies have questioned the significance of OM for patient outcomes in PS patients [16,18]. 

Consequently, this study aims to address this gap in evidence through a retrospective analysis of a consistent cohort of PS patients. The cohort includes patients managed with a multistage wound protocol, with a focus on the prevalence of OM, as well as clinical characteristics and blood markers indicative of infection.

## 2. Materials and Methods

### 2.1. Study Design and Data Collection

**Figure 1:**
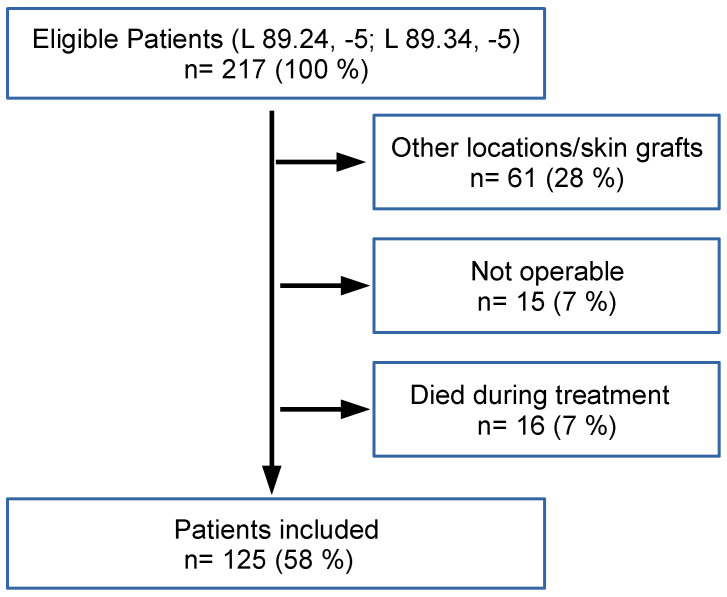
Flow chart showing the recruitment and the exclusion criteria. Of the 217 patients screened, 92 had to be excluded due to the reasons above.

Institutional research ethics committee approval (Ref. 16-294-101) was obtained prior to the initiation of this study. Adhering to the Standards for Reporting Diagnostic Accuracy Studies (STARD) guidelines, a retrospective chart review was conducted for patients undergoing surgical therapy for the management of ischial, sacral, and trochanteric pressure sores (PS) at the study center between January 2014 and June 2019. The patients were identified using an initial ICD-10-GM-2022 code-based search (L 89.24, −5; L 89.34, −5) of the institutional administrative database. Exclusion criteria included patients treated solely by primary closure, skin grafting, or surgical debridement only, non-operable patients, and those who died during treatment (Figure 1). 

Patient records were screened for demographic data, comorbidities (e.g., paraplegia, neurodegenerative disorders, fecal incontinence/presence of a colostomy, diabetes, and BMI (kg/m^2^)), medication, mobility status, and other factors potentially predisposing them to the development of PS. PS characteristics such as location, size (Diameter Small: <5 cm; Medium: 5–10 cm; Large: >10 cm), and distance to the anus were recorded at admission. Operative and postoperative details, including the total number of surgical debridement, major complications requiring surgical revisions, and length of hospital stay (LOS), were also documented. Laboratory parameters relevant for detection of infection and kidney function, collected on admission, were c-reactive protein (CRP in mg/dL), leukocyte count (*1000/µL), hemoglobin level (g/dL), and renal retention parameters (creatinine in mg/dL), with glomerular filtration rate (GFR in mL/min).

### 2.2. Treatment Protocol 

Before flap reconstruction, wound conditioning was achieved through serial debridement and the application of negative pressure wound therapy (NPWT). Serial debridement consisted of at three sessions involving the surgical removal of necrotic tissue within PS. NPWT was applied in between debridement sessions with a standard setting of 125 mmHg suction [10,19]. A multi-disciplinary team, including specialists from internal medicine, plastic surgery, wound care management, physical rehabilitation, and social services, carefully coordinated care (see also Supplemental Materials “Treatment Algorithm”). 

### 2.3. Microbiological Processing and Histopathological Analysis

Intraoperative biopsies were collected with a rongeur and sent in for microbiological incubation and histopathological evaluation. Osteomyelitis (OM) was defined by the presence of lymphocytes and plasma cells, with or without neutrophils, in bone biopsies [18]. Germ detection was documented following a 5-day incubation period, adhering to in-house standards.

### 2.4. Statistical Analysis

Percentages represent the corresponding fractions in brackets. Continuous variables were summarized with mean and standard deviation, and differences were analyzed using Student’s *t*-test. Non-parametric variables were summarized with median and interquartile range, and differences among groups were tested according to Mann–Whitney U and Kruskal–Wallis tests. Categorical variables were compared using Chi-square and Fisher’s exact tests. Regression analysis was not performed due to the inconsistency of OM as an independent variable. The most homogenous subgroup was the consistent OM-positive group (three positive bone biopsies). The most homogeneous subgroup was the consistent OM-positive group (three positive bone biopsies). However, due to the small sample size, covariate analysis and regression models were not suitable. Instead, associations were described descriptively. *p*-values < 0.05 were considered statistically significant. Statistical analyses were performed using IBM SPSS Version 27.0 (IBM Corp., Armonk, NY, USA). 

## 3. Results

### 3.1. Patient Cohort

Table 1 provides an overview of the patient demographics. No significant disparities were observed overall and between the groups in terms of sex, reason of bedrest, region of PS, age, and BMI (Table 1 and Table 2). Overall, OM was histologically diagnosed in 74 cases (74/125, 59%). In 29 of the 51 cases (57%) with no OM detection, there was insufficient or no bone substance available for histological examination.

### 3.2. OM-Positive vs. OM-Negative Biopsies

**Figure 2:**
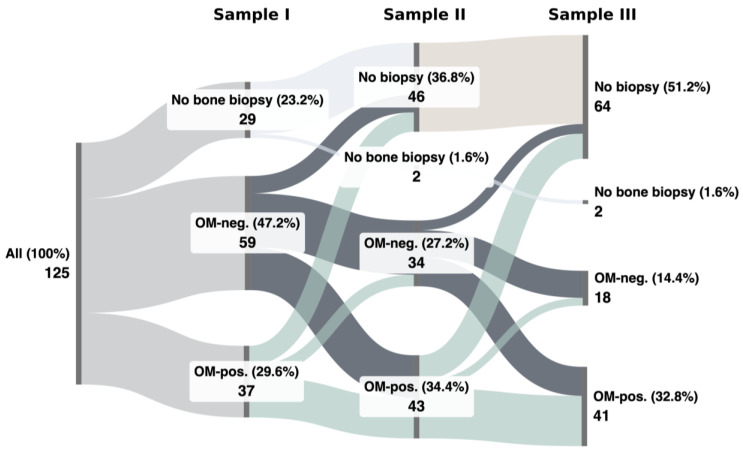
This Sankey plot visualizes the detection osteomyelitis-positive patients (OM-pos.) or absence of osteomyelitis (OM-neg.) in biopsies taken during the first (I), second (II), and third (III) debridement procedures.

Figure 2 illustrates the progression of histological findings across various biopsy intervals. Notably, as the number of debridement procedures increased, there was a corresponding rise in the prevalence of OM.

Remarkably, when biopsies lacking bone tissue are excluded from the analysis, OM was detected in only 39% (37/96) of the samples taken during the first debridement session. A progression in the prevalence of OM was observed, increasing to 56% (43/77) at the second debridement interval and even further to 70% (41/59) at the third debridement. The proportion of patients in whom no OM was detected, whether in all three biopsies or at least one, is low with 22 cases (22/125, 18%). In 11 cases (11/125, 9%), all three biopsies showed no signs of OM (no OM). OM was consistently diagnosed in all three debridement procedures in 15 (15/125, 12%) cases. Ten patients (10/125, 9%) presented temporary diagnosis of OM. In 35 cases (35/125, 28%), no OM was present in the first biopsy, but manifested in the subsequent debridement. 

In cases with histologic evidence of OM, the depth and size of the PS were notably greater in the OM-positive group (Table 1). Additionally, patients in the OM-positive group underwent a higher frequency of debridement sessions (Table 1) and the duration of hospitalization was significantly longer (Table 2).

**Figure 3:**
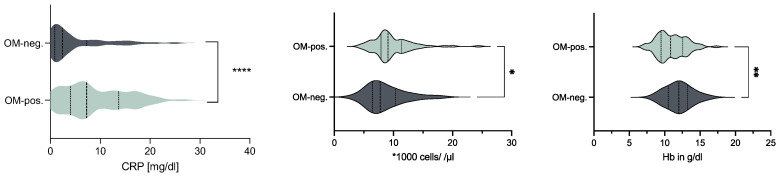
Blood markers of enrolled patients upon admission. Levels of C-reactive protein (CRP) (*p* < 0.001) and leukocyte count (*p* = 0.028) were significantly higher in osteomyelitis-positive patients (OM-pos.), whereas hemoglobin levels (Hb) were significantly lower (*p* < 0.004). (* *p* < 0.05, ** *p* < 0.005 and **** *p* < 0.0001).

Patients diagnosed with OM had significantly higher blood markers indicative of infection upon admission, including elevated c-reactive protein (CRP) levels (*p* < 0.001) and increased leukocyte counts (*p* < 0.028). Additionally, these patients showed reduced hemoglobin counts (*p* < 0.004) (Figure 3). No differences were observed in the nephrological parameters (Table 2).

### 3.3. Consistent OM-Positivity vs. Consistent OM-Negativity

In a subgroup analysis, patients consistently diagnosed with OM across three histological examinations (*n* = 15) had a significantly longer median length of stay (LOS) compared to those consistently negative for OM (*n* = 11) (44 days vs. 28 days, *p* = 0.015). However, no significant differences were observed in infection parameters between the groups (Table 3).

Consistently, OM-positive patients required significantly more flap revisions. Although not significant, there was trend towards more OM-positive patients discharged with impaired wound healing (Table 4).

### 3.4. Microbiological Findings

Fourteen patients (14/125, 11%) required isolation upon admission due to colonization by specific multidrug-resistant organisms identified in previous hospitalizations. During hospitalization, isolation was required more frequently in the OM-positive group (12/125, 10%) compared to the OM-negative group (7/125, 6%). 

Antibiotics were administrated in 107 cases (107/125, 86%), with the OM-positive group receiving antibiotics in 93% of the cases (69/74) compared to the OM-negative group receiving antibiotics in 75% of the cases (38/51). 

**Figure 4:**
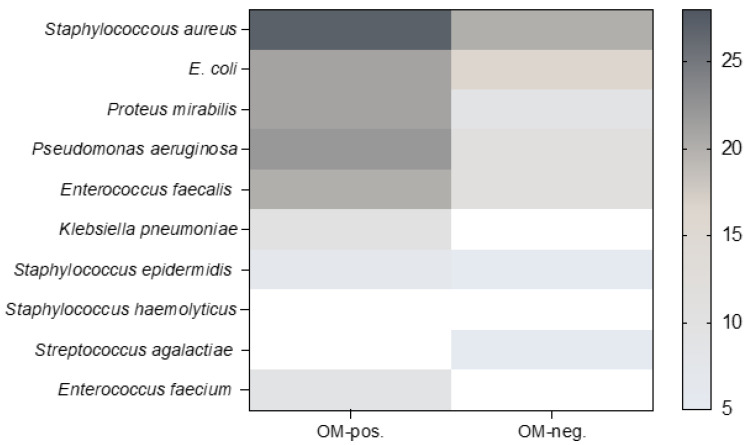
Plot displays bacterial species identified in PS of OM-positive and OM-negative patients. No significant accumulation of specific pathogen species was observed in either of the two groups (*n* = 125).

Regarding the multi bacterial species within the wound flora, we were unable to link OM-positivity to specific pathogens (Figure 4).

## 4. Discussion

Successful treatment of infected PS is challenging and often requires multiple surgical interventions, which can be highly burdensome for the patient [5,17]. In late-stage PS, the risk of osteomyelitis (OM) is particularly high due to exposed bone [15]. Consequently, in this cohort, histological positivity for OM was associated with an increasing PS lesion stage and diameter (Table 1). 

Although imaging tools such as magnetic resonance imaging (MRI) and positron emission tomography-computed tomography (PET-CT) are recognized methods for screening for OM, their low specificity makes bone biopsies the gold standard for confirming the diagnosis and identifying the specific organism causing the infection [12,13,14]. Brunel et al. conducted a prospective trial in 34 patients with pelvic PS, and suggested a multiple-biopsies strategy of at least three bone samples to reliably detect pathogens in PS associated OM [13]. In this study, histological evidence for OM was found in 59% of the PS patients (74/125) but only 15 patients (12%, 15/125) had consistent-OM with three positive biopsies (Table 4). Of note, in half of these OM-positive cases (35/125, 28%), OM was not evident in the bone samples taken during the index procedure (Figure 2). 

OM is defined by histological changes in bone tissue, including the presence of lymphocytes and plasma cells with or without neutrophils [18]. However, these changes can be detected in PS lesions of all stages, reflecting the natural tissue response to inflammation and mechanical stress [18]. Although the additional presence of pathogens may further support the suspicion for bacterial OM, distinguishing colonization from infection in polymicrobial contaminated ulcers is challenging and may lead to an overestimation of bacterial infection rates [20,21]. In fact, a previous histopathologic study in PS affected pelvic bone has demonstrated that sacral bone sections typically exhibit bone destruction, repair response, and occasionally superficial chronic and acute OM, but of note, histological signs of severe OM in PS lesions seem to be surprisingly rare [18]. 

The process of bone biopsy extraction inevitably disrupts cortical bone, increasing its vulnerability to contamination during serial debridement and sample collection. Hence, secondary contamination during the phase of serial debridement and sample collections could be a plausible explanation for the significant increase in OM-positive bone biopsies observed. In contrast, other authors found serial debridement of PS to be safe, and suggested a prevention of sepsis and death in PS patients with multiple co-morbid conditions [6,8,22]. More importantly, the implications of confirmed OM in PS patients have been questioned previously [16,18]. When Larson et al. investigated a cohort of 111 PS patients, no correlation was found between positive bone cultures and recurrence or complication rates [16]. In our cohort, only the consistent OM-positive patients (three positive biopsies) required significantly more flap revisions, corresponding to a longer LOS (Table 3 and Table 4). However, a probable confounder might be that small- and medium-sized PS, which have been demonstrated to generally facilitate uncomplicated wound healing, are clustered in the OM-negative group (Table 1) [5]. Therefore, this finding is more likely to be coincidental than indicative of any correlation. Regardless, Larson et al. advocated for immediate soft tissue coverage irrespective of the bone culture results, and thereby discouraged established multistep debridement protocols prior to reconstruction. This is line with additional studies in this field that were unable to detect any correlation between histologically proven OM and patient outcome [15,17]. 

These previous observations suggest that the clinical pathogenicity of microorganisms in highly contaminated PS might be generally overrated when compared to outcomes seen in long bone infections associated with implants or fractures [4]. Perhaps the trabecular microenvironment of cancellous pelvic bone, known to be rich in inflammatory cells, is less susceptible to exogenous infections than the less vascularized compact cortical bone structure of the lower extremity [23]. Moreover, existing recommendations for antibiotic therapy for OM-positive PS vary considerably from as short as 5 days to 6 weeks. Remarkably, so far, no correlation has been found between recurrence rates and the duration of antibiotic therapy [15,17]. The transition from micro-organism colonization and infection in chronic wounds, such as PS lesions, and its consequences for impaired wound healing are still widely unclear. Indeed, a Cochrane review analyzing randomized controlled trials on the effects of antibiotics and antiseptics on wound healing in PS found weak evidence in favor of the comparator treatment without antimicrobial properties [21]. Correspondingly, this investigation identified a trend toward a higher number of constant OM-positive patients (three positive biopsies) being discharged with impaired wound healing, without reaching statistical significance (Table 4).

Given the lack of association between histologically verified OM and adverse clinical outcomes, and considering the increasing prevalence of multidrug-resistant organisms, the primary rationale for antibiotic management in PS patients should focus on treating existing sepsis [15,16,17,20,21]. This is consistent with highly significant elevations of C-reactive protein levels and significant changes in blood counts detected in patients with positive histological evidence of OM in this cohort (Figure 3). In fact, previous investigations have discouraged routine collection of bone biopsies in the absence of sepsis, based on the generally mild histological bone involvement even in late-stage PS lesions [18]. The findings of this study support a more cautious approach to managing OM in PS patients, emphasizing the importance of clinical judgment over routine bone biopsies in the absence of sepsis.

Nevertheless, the specific cause for the relatively low impact of diagnosed OM for the clinical outcome in PS patients remains unclear. Although the organisms cultured in this cohort resemble organisms in post-traumatic OM, biofilm formation and its associated resistance to antimicrobial factors seems to be of minor clinical relevance in PS patients (Figure 4) [15,17,21,24]. One reason for this difference in patient outcomes between post-traumatic and PS-associated OM could be the absence of implant-related complications in PS cohorts. On the other hand, it is important to emphasize that wound dehiscence rates are exceptionally high in PS patients. Determining whether mechanical forces or tissue infection is the predominant cause of this complication is challenging, especially under the aspect that the spectrum of pathogens may change within the same patient. Jugun et al. analyzed a cohort of PS patients with a recurrence rate of 63% and found a different spectrum of pathogens in 86% of PS recurrences compared to the initial hospitalization episode [17]. 

In this cohort, the detected wound flora contained multiple pathogens in all the patients, whereas *Staphylococcus aureus* and Gram-negative bacteria clearly dominated in the microbial colonization (Figure 4). Nevertheless, this multi-microbial colonization made it impossible to determine which specific microorganism might predominate, leading to negative outcomes. Other observations suggested a mean number of 5.8 species per PS in the presence of necrotic tissue [25]. Remarkably, no obvious relationship between the density of microorganisms and the eventual outcome of the myocutaneous flap procedure existed [25]. This underlines the complexity of the interplay of host response to bioburden and the ideal environment for microbial colonization in PS lesions. 

The retrospective design of this trial imposes certain limitations. While findings from a single-center trial may not generalize in every aspect beyond the specific patient population, the case volume of this study remains substantial and of high consistency. Although secondary contamination is one reasonable explanation of the rise of OM-positive samples, the causality of this observation remains unproven. Other confounding variables, such as the selection of biopsy site, could have influenced the detection of OM, which remains an uncontrolled factor due to the retrospective nature of these data. However, multiple uncertainties of OM in the context of PS treatment exist, and despite the substantial burden of disease and financial expenses, the influence of numerous factors in unsuccessful PS treatment challenge any attempt to evidence-based decision-making. Considering the discussed previous studies in this context and the limited impact of OM on the outcomes in this cohort, the findings from this trial provide support for adopting a more prudent strategy in managing OM in PS patients.

While this study did not find a significant association between OM and clinical outcomes, it is important to consider the general limitations of a retrospective analysis. Further prospective studies are necessary to validate these findings and investigate the potential advantages of adopting more selective biopsy protocols. Future research could focus on identifying specific biomarkers that can predict OM with greater accuracy in PS patients, thereby decreasing the necessity for invasive diagnostic procedures. Given that PS care represents a significant cost driver and places substantial individual burdens, there persists a notable gap in evidence-based preventive measures in this area, which should also be addressed going forward.

## 5. Conclusions

In summary, this study highlights the complex nature of OM in PS conditions. Despite this, consistent with previous investigations, we did not find compelling evidence linking histologically proven OM with adverse clinical outcomes in PS patients. This raises fundamental questions about the clinical significance of OM in the context of PS treatment. The lack of a clear link between OM and clinical outcomes suggests that the routine collection of bone biopsies may not be indicated in all PS patients. In fact, uncritical bone biopsy collection may facilitate the transmission of multibacterial wound flora, potentially leading to OM during serial debridement.

In the absence of elevated inflammatory markers, reducing frequency of bone biopsies and adopting a less aggressive approach to bone debridement may be crucial for preventing OM in PS patients. This finding aligns with existing evidence questioning the utility of aggressive diagnostic procedures in this patient population. Under the aspect of PS care being among the main cost drivers and, more importantly, the substantial individual burden associated with PS management, there is a notable imbalance for preventive measures in this field.

## Figures and Tables

**Table 1:** Patient characteristics of the entire cohort (All), osteomyelitis-negative (OM-neg.), and osteomyelitis-positive (OM-pos.) patients.

		All (*n* = 125)	OM-neg. (*n* = 51)	OM-pos. (*n* = 74)	*p*
Sex	Female	57 (45.6%)	23 (45.1%)	34 (45.9%)	0.925
	Male	68 (54.4%)	28 (54.9%)	40 (54.1%)	
Reason for bedrest	Frailty	24 (19.2%)	11 (21.6%)	13 (17.6%)	0.683
	Paraplegia	42 (33.6%)	15 (29.4%)	27 (36.5%)	
	Other	59 (47.2%)	25 (49%)	34 (45.9%)	
Region	Sacral	80 (64.0%)	29 (56.9%)	51 (68.9%)	0.200
	Ischial	34 (27.2%)	15 (29.4%)	19 (25.7%)	
	Trochanteric	11 (8.8%)	7 (13.7%)	4 (5.4%)	
Distance to anus	<3 cm	27 (21.6%)	10 (19.6%)	17 (23%)	0.376
	>3 cm	79 (63.2%)	37 (72.5%)	42 (56.8%)	
	Missing	19 (15.2%)	4 (7.8%)	15 (20.2%)	
Colostomy	None	95 (76.0%)	45 (88.2%)	50 (67.2%)	**0.014**
	Present at admission	23 (18.4%)	6 (11.8%)	17 (23%)	
	Newly applied	7 (0%)	0 (0%)	7 (9.8%)	
Grading	3	25 (20.2%)	20 (39.2%)	5 (6.8%)	**<0.001**
	4	100 (79.8%)	31 (60.8%)	69 (93.2%)	
Area	Small (<5 cm)	53 (42.4%)	34 (66.7%)	19 (26.8%)	**<0.001**
	Medium (5–10 cm)	50 (40.0%)	15 (29.4%)	35 (49.3%)	
	Large (>10 cm)	19 (15.2%)	2 (3.9%)	17 (23.9%)	
	Missing	3 (2.4%)	0 (0%)	3 (4.1%)	
Histologic samples	1	33 (26.4%)	23 (45.1%)	10 (13.5%)	**<0.001**
	2	21 (16.8%)	5 (9.8%)	16 (21.6%)	
	3	71 (56.8%)	23 (45.1%)	48 (64.9%)	

**Table 2:** Patient characteristics. (BMI: body mass index; LOS: length of hospital stay; GFR: glomerular filtration rate).

	Total (*n* = 125)	OM-neg. (*n* = 51)	OM-pos. (*n* = 74)	*p*
Age (years)	66 (13–95)	66 (13–95)	67 (16–95)	0.714
BMI (kg/m^2^)	24.9 ± 6.9 (*n* = 121)	24.8 ± 7.4 (*n* = 49)	23.8 ± 6.5 (*n* = 71)	0.804
LOS (days)	27 (6–119)	22 (6–98)	33 (15–119)	**<0.001**
Creatinine (mg/dL)	0.7 (0.09–2.73)	0.69 (0.09–2.63)	0.68 (0.24–2.73)	0.888
GFR (mL/min)	101 (17–140)	101 (17–140)	99 (18–140)	0.909

**Table 3:** Characteristics of patients either consistently osteomyelitis-positive (OM-pos.) or consistently osteomyelitis-negative (OM-neg.). (BMI: body mass index; LOS: length of hospital stay; CRP: C-reactive protein; HB: hemoglobin level; GFR: glomerular filtration rate).

	Consistent OM-neg. (*n* = 11)	Consistent OM-pos. (*n* = 15)	*p*
Age (years)	64 (14–91)	65 (27–89)	0.54
BMI (kg/m^2^)	23.2 ± 5.1	24.5 ± 4.9	0.344
LOS (days)	28 (16–42)	44 (15–119)	**0.015**
CRP (mg/dL)	4.2 (0.32–18)	10.1 (1.75–21.9)	0.144
Leukocytes (*1000/µL)	7.7 (3–17.3)	8.6 (6.2–18.6)	0.474
HB (g/dL)	13.3 (8.5–15.7)	10.3 (8.5–15.2)	0.077
Creatinine (mg/dL)	0.72 (0.39–2.5)	0.71 (0.31–2.73)	0.878
GFR (mL/min)	99 (17–140)	100 (18–140)	1

**Table 4:** Complication rates of patients either consistently positive (OM-pos.) or consistently negative (OM-neg.) for osteomyelitis.

		Consistent OM-pos. (*n* = 15)	Consistent OM neg. (*n* = 11)	*p*
Flap revision	No revision	7 (46.7%)	11 (100%)	**0.007**
	Revision	8 (53.3%)	0 (0%)	
Wound status at discharge	Closed	12 (80%)	10 (90.9%)	0.614
	Open	3 (20%)	1 (9.1%)	

## Data Availability

All data were presented in this paper. Inquiries regarding the raw data can be addressed to the corresponding author at any time.

## Supplementary Materials

The following supporting information can be downloaded at: https://www.mdpi.com/article/10.3390/life14080973/s1, File S1. Treatment Algorithm.

## References

[1] Gillespie B.M., Chaboyer W.P., McInnes E., Kent B., A Whitty J., Thalib L. (2014). Repositioning for pressure ulcer prevention in adults. Cochrane Database Syst. Rev..

[2] Kottner J., Cuddigan J., Carville K., Balzer K., Berlowitz D., Law S., Litchford M., Mitchell P., Moore Z., Pittman J. (2020). Pressure ulcer/injury classification today: An international perspective. J. Tissue Viability.

[3] Bialowas C., Nguyen B., Patel A.M. (2021). Best Solutions for Perineal and Pressure Sore Reconstruction. Plast. Reconstr. Surg..

[4] Lew D.P., Waldvogel F.A. (2004). Osteomyelitis. Lancet.

[5] Anker A.M., Ruewe M., Prantl L.M., Geis S., Kehrer A., Baringer M., Schiltz D., Zeman F.M., Vykoukal J., Klein S.M. (2022). “A-PePSI LIGhT” Assessment Score to Predict Pressure Sore Impaired Healing Late Recurrence, Immobility, Greater Surface, Inhibited Thrombocytes. Plast. Reconstr. Surg..

[6] Suissa D., Danino A., Nikolis A. (2011). Negative-pressure therapy versus standard wound care: A meta-analysis of randomized trials. Plast. Reconstr. Surg..

[7] Moog P., Jensch M., Betzl J., Bauer A.-T., Cerny M.K., Schmauss D., Kükrek H., Erne H., Machens H.-G., Megerle K. (2021). Bacterial bioburden of wounds: Influence of debridement and negative-pressure wound therapy (NPWT). J. Wound Care.

[8] Schiffman J., Golinko M.S., Yan A., Flattau A., Tomic-Canic M., Brem H. (2009). Operative debridement of pressure ulcers. World J. Surg..

[9] Rennert R., Golinko M., Yan A., Flattau A., Tomic-Canic M., Brem H. (2009). Developing and evaluating outcomes of an evidence-based protocol for the treatment of osteomyelitis in Stage IV pressure ulcers: A literature and wound electronic medical record database review. Ostomy Wound Manag..

[10] Dumville J.C., Webster J., Evans D., Land L. (2015). Negative pressure wound therapy for treating pressure ulcers. Cochrane Database Syst. Rev..

[11] Wassif R.K., Elkayal M., Shamma R.N., Elkheshen S.A. (2021). Recent advances in the local antibiotics delivery systems for management of osteomyelitis. Drug Deliv..

[12] Wong D., Holtom P., Spellberg B. (2019). Osteomyelitis Complicating Sacral Pressure Ulcers: Whether or Not to Treat with Antibiotic Therapy. Clin. Infect. Dis..

[13] Brunel A.-S., Lamy B., Cyteval C., Perrochia H., Téot L., Masson R., Bertet H., Bourdon A., Morquin D., Reynes J. (2016). Diagnosing pelvic osteomyelitis beneath pressure ulcers in spinal cord injured patients: A prospective study. Clin. Microbiol. Infect..

[14] Chicco M., Singh P., Beitverda Y., Williams G., Hirji H., Rao G.G. (2020). Diagnosing pelvic osteomyelitis in patients with pressure ulcers: A systematic review comparing bone histology with alternative diagnostic modalities. J. Bone Jt. Infect..

[15] Marriott R., Rubayi S. (2008). Successful Truncated Osteomyelitis Treatment for Chronic Osteomyelitis Secondary to Pressure Ulcers in Spinal Cord Injury Patients. Ann. Plast. Surg..

[16] Larson D.L.M., Hudak K.A.M., Waring W.P.M., Orr M.R.M., Simonelic K.M. (2012). Protocol Management of Late-Stage Pressure Ulcers: A 5-Year Retrospective Study of 101 Consecutive Patients with 179 Ulcers. Plast. Reconstr. Surg..

[17] Jugun K., Richard J.-C., Lipsky B.A.M., Kressmann B., Pittet-Cuenod B., Suvà D., Modarressi A., Uçkay I. (2016). Factors Associated with Treatment Failure of Infected Pressure Sores. Ann. Surg..

[18] Türk E.E., Tsokos M., Delling G. (2003). Autopsy-Based Assessment of Extent and Type of Osteomyelitis in Advanced-Grade Sacral Decubitus Ulcers: A Histopathologic Study. Arch. Pathol. Lab. Med..

[19] Arowojolu O.A., Wirth G.A.M. (2021). Sacral and Ischial Pressure Ulcer Management with Negative-Pressure Wound Therapy with Instillation and Dwell. Plast. Reconstr. Surg..

[20] Cataldo M.C., Bonura C., Caputo G., Aleo A., Rizzo G., Geraci D.M., Calà C., Fasciana T., Mattaliano A.R., Mammina C. (2011). Colonization of pressure ulcers by multidrug-resistant microorganisms in patients receiving home care. Scand. J. Infect. Dis..

[21] Norman G., Dumville J.C., Moore Z.E., Tanner J., Christie J., Goto S. (2016). Antibiotics and antiseptics for pressure ulcers. Cochrane Database Syst. Rev..

[22] Braga I.A., Pirett C.C.N.S., Ribas R.M., Filho P.G., Filho A.D. (2013). Bacterial colonization of pressure ulcers: Assessment of risk for bloodstream infection and impact on patient outcomes. J. Hosp. Infect..

[23] Khan S.N., Cammisa F.P., Sandhu H.S., Diwan A.D., Girardi F.P., Lane J.M. (2005). The biology of bone grafting. J. Am. Acad. Orthop. Surg..

[24] Shah N.S., Kanhere A.P., Dowell E., Sabbagh R.S., Bonamer J., Franklin A., Sanders D.T., Sagi H.C. (2023). Risk Factors and Characteristics of Recalcitrant Osteomyelitis After Initial Surgical and Antibiotic Treatment. J. Orthop. Trauma..

[25] Sapico F.L., Ginunas V.J., Thornhill-Joynes M., Canawati H.N., Capen D.A., Klein N.E., Khawam S., Montgomerie J.Z. (1986). Quantitative microbiology of pressure sores in different stages of healing. Diagn. Microbiol. Infect. Dis..

