# Supplementary figures and images for: Osteomyelitis in Late-Stage Pressure Sore Patients: A Retrospective Analysis
Source: Life (Basel). 2024 Aug 2;14(8):973. doi: 10.3390/life14080973 (PMC11355209; doi:10.3390/life14080973)

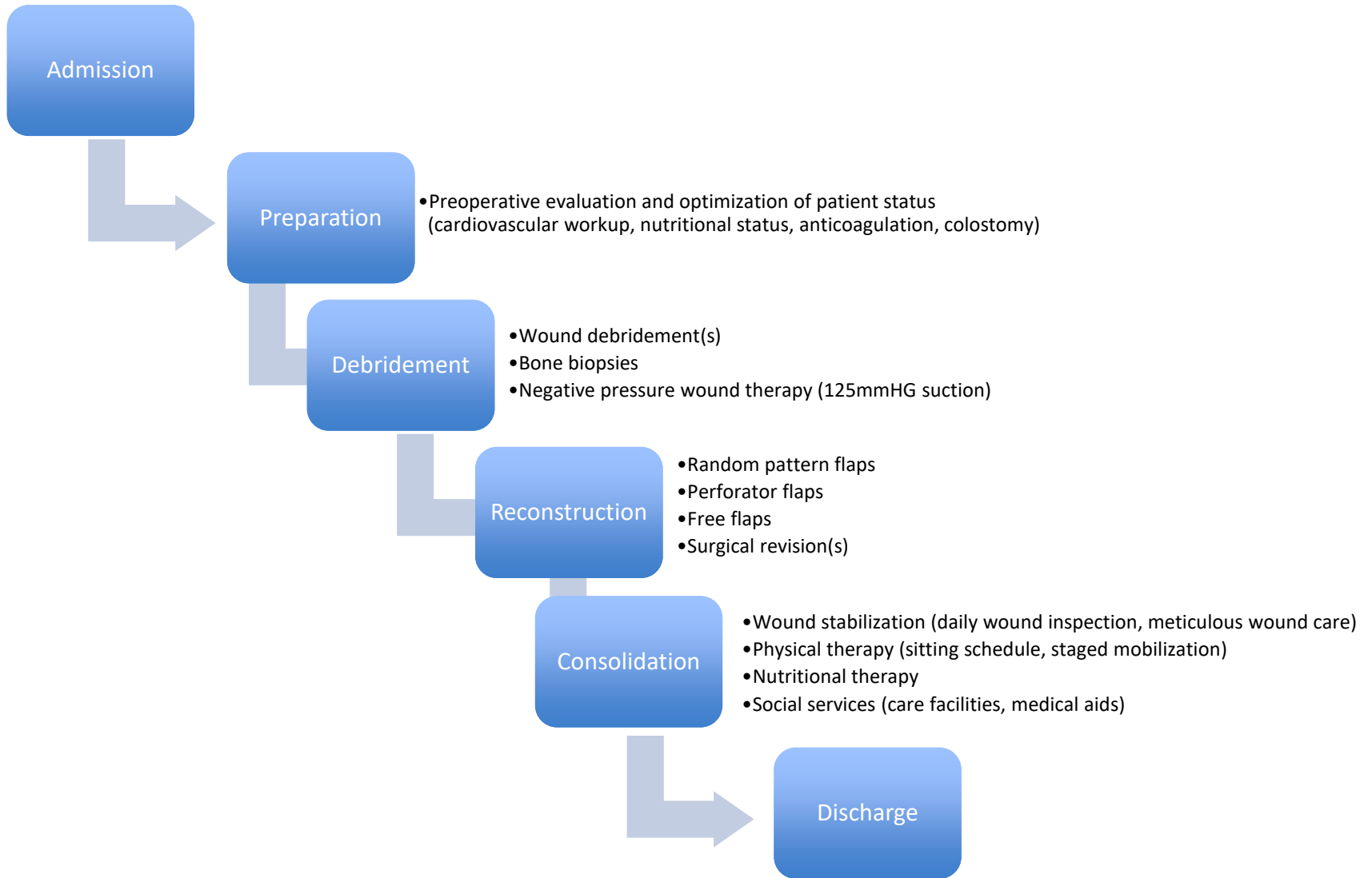

Supplement: Supplementary file 1 [file life-14-00973-s001.zip › life-3060154-supplementary.pdf]
